# Predominantly positive XCO_2_ anomalies in the Caatinga biome highlight carbon vulnerability

**DOI:** 10.1038/s41598-026-37629-1

**Published:** 2026-02-08

**Authors:** Libério Junio Silva, Luis Miguel da Costa, Ricardo de Oliveira Bordonal, Alan Rodrigo Panosso, Thiago Torres Costa Pereira, Cassiano Gustavo Messias, Newton La Scala

**Affiliations:** 1 Graduate Program in Environmental Sciences, State University of Minas Gerais, Av. Professor Mário Palmério, 1001–Universitário District, Frutal, Brazil; 2 Department of Engineering and Exact Sciences, São Paulo State University (FCAV–UNESP), Via de Acesso Prof. Paulo Donato Castellane S/n, Jaboticabal, 14884-900 Brazil; 3 Brazilian Biorenewables National Laboratory/Brazilian Center for Research in Energy and Materials (LNBR/CNPEM), Campinas, São Paulo Brazil; 4 General Coordination for Land Sciences (CGCT), National Institute for Space Research (INPE), Av. dos Astronautas, 1758, Jardim da Granja, São José dos Campos, SP 12227-010 Brazil

**Keywords:** Caatinga biome, Carbon cycle, OCO-2 satellite, XCO_2_ anomalies, Climate variability., Climate sciences, Ecology, Ecology, Environmental sciences

## Abstract

The Caatinga biome, the only exclusively Brazilian biome, plays a crucial yet understudied role in regional and global carbon dynamics. Using column-averaged dry-air mole fraction of CO_2_ (XCO_2_) data from NASA’s Orbiting Carbon Observatory-2 (OCO-2) between 2015 and 2022, this study investigates spatial and temporal anomalies across distinct phytoecological biozones of the Caatinga. Anomaly detection, spatial autocorrelation (Local Moran’s I), time-series modeling (ARIMA), and correlation analyses with vegetation and climate indices (NDVI, EVI, LAI, land surface temperature, and precipitation) were applied to evaluate the biome’s carbon balance. Results reveal heterogeneous XCO_2_ patterns, with predominantly negative or neutral anomalies, confirming the Caatinga’s role as a carbon sink, though punctuated by localized positive anomalies indicating emission hotspots. The Savanna-Steppe and Pioneer Formation biozones exhibited the strongest seasonal and spatial clustering of positive anomalies, highlighting vulnerability to land-use pressures and climatic extremes. Forested biozones, particularly Open and Dense Ombrophilous Forests, showed increasing anomaly trends in recent years, suggesting a potential weakening of sink capacity. Correlations revealed distinct biome-specific responses: positive associations between XCO_2_ and precipitation in transitional and pioneer formations, and negative associations with vegetation indices in savanna areas, emphasizing hydrological control of carbon fluxes. The findings demonstrate that the Caatinga exhibits both resilience and vulnerability, with its carbon balance strongly modulated by climatic variability, vegetation structure, and anthropogenic pressures. These results underscore the biome’s strategic role in climate mitigation and the urgent need for targeted conservation and restoration policies to safeguard its carbon sequestration potential.

## Introduction

 Global climate change is one of the greatest challenges of our time, deeply affecting natural systems, societies, and economies^1–4^. A key driver of this phenomenon is the increasing atmospheric concentration of greenhouse gases (GHGs), particularly carbon dioxide (CO_2_), primarily fueled by the combustion of fossil fuels and, in tropical countries such as Brazil, by land-use and land-cover changes, especially deforestation^5–8^.

In this context, orbital sensors have emerged as strategic tools for monitoring the atmospheric distribution of CO_2_ and understanding the global carbon cycle, especially in remote and hard-to-access areas^9–12^. Satellites such as GOSAT (Greenhouse Gases Observing Satellite), developed by JAXA, and NASA’s OCO-2 and OCO-3 (Orbiting Carbon Observatory) were specifically designed to quantify the column-averaged dry-air mole fraction of CO_2_ (XCO_2_), allowing for the investigation of its spatial and temporal variability^12–14^.

Most XCO_2_-related studies have focused on the Amazon due to its relevance for global carbon removals and its role in climate regulation^15,16^. However, other Brazilian regions, such as the semi-arid Northeast, remain underrepresented in such analyses. The Caatinga biome, exclusively Brazilian and covering approximately 10% of the national territory, presents a unique combination of ecological, climatic, and socio-environmental characteristics, making it a strategic region for studying the interactions among carbon, climate, and human activity^11,12^. Nevertheless, the behavior of XCO_2_ in the Caatinga remains poorly understood.

Investigating the spatial and temporal variability and anomalies of XCO_2_ in the Caatinga can provide valuable insights into the regional carbon balance and the biome’s response to natural and anthropogenic disturbances. Decreases in XCO_2_ levels typically indicate carbon uptake by vegetation, while increases may signal emissions associated with processes such as environmental degradation or climatic variability^13,17–19^. Recent studies have shown that negative XCO_2_ anomalies are linked to ecological variables such as gross primary production (GPP) and solar-induced chlorophyll fluorescence (SIF), underscoring the potential of these data for biophysical inference^14,20^.

Moreover, spatial variations in XCO_2_ can reflect emissions derived from fossil fuel combustion, land use, and urban expansion^21–24^, thus serving as valuable indicators for identifying strategic mitigation efforts^25,26^. These patterns have encouraged the development of inverse modeling approaches to estimate emissions based on atmospheric observations^13,26,27^.

Despite the advantages of satellite-derived XCO_2_ observations for global and regional CO_2_ monitoring, retrieval accuracy can be adversely affected by surface and atmospheric conditions. Reports indicate that uncertainties may increase in regions with high surface reflectance, sparse vegetation, intense aerosol loads, and heterogeneous land cover characteristics commonly found in drylands such as the Caatinga. These factors can lead to spatial and temporal biases in XCO_2_ estimates, highlighting the need for regional assessments and careful interpretation of satellite-derived data in semi-arid environments^28,29^.

Additionally, radiometric performance degradation has been documented in long-term satellite-based products used to infer biophysical responses to atmospheric CO_2_, such as those derived from MODIS. Although MODIS vegetation indices remain some of the most widely used Earth observation products due to their global coverage and temporal consistency, studies have revealed sensor aging effects and calibration drifts that may influence data stability over extended time periods (e.g., Lyapustin et al., 2014^30^. Therefore, integrating XCO_2_ observations with complementary variables should incorporate considerations of long-term sensor uncertainties to ensure robust environmental assessments.

In light of this context, the present study aims to investigate XCO_2_ anomalies in the Caatinga biome using data from the OCO-2 satellite, considering its distinct phytoecological zones, here referred to as biozones: *Contact areas (Ecotone-Enclave)*, *Deciduous Seasonal Forest*,* Semideciduous Seasonal Forest*,* Savanna*,* Steppe-Savanna*,* Open Ombrophilous Forest*,* Pioneer Formation*, and *Dense Ombrophilous Forest*. By mapping and analyzing XCO_2_ patterns across these biozones, we aim to address important gaps in the understanding of the carbon cycle in Brazil’s semi-arid regions and contribute to the broader knowledge of CO_2_ emission and sequestration mechanisms under extreme environmental conditions and increasing anthropogenic pressure.

## Method

### Study area

**Fig. 1:**
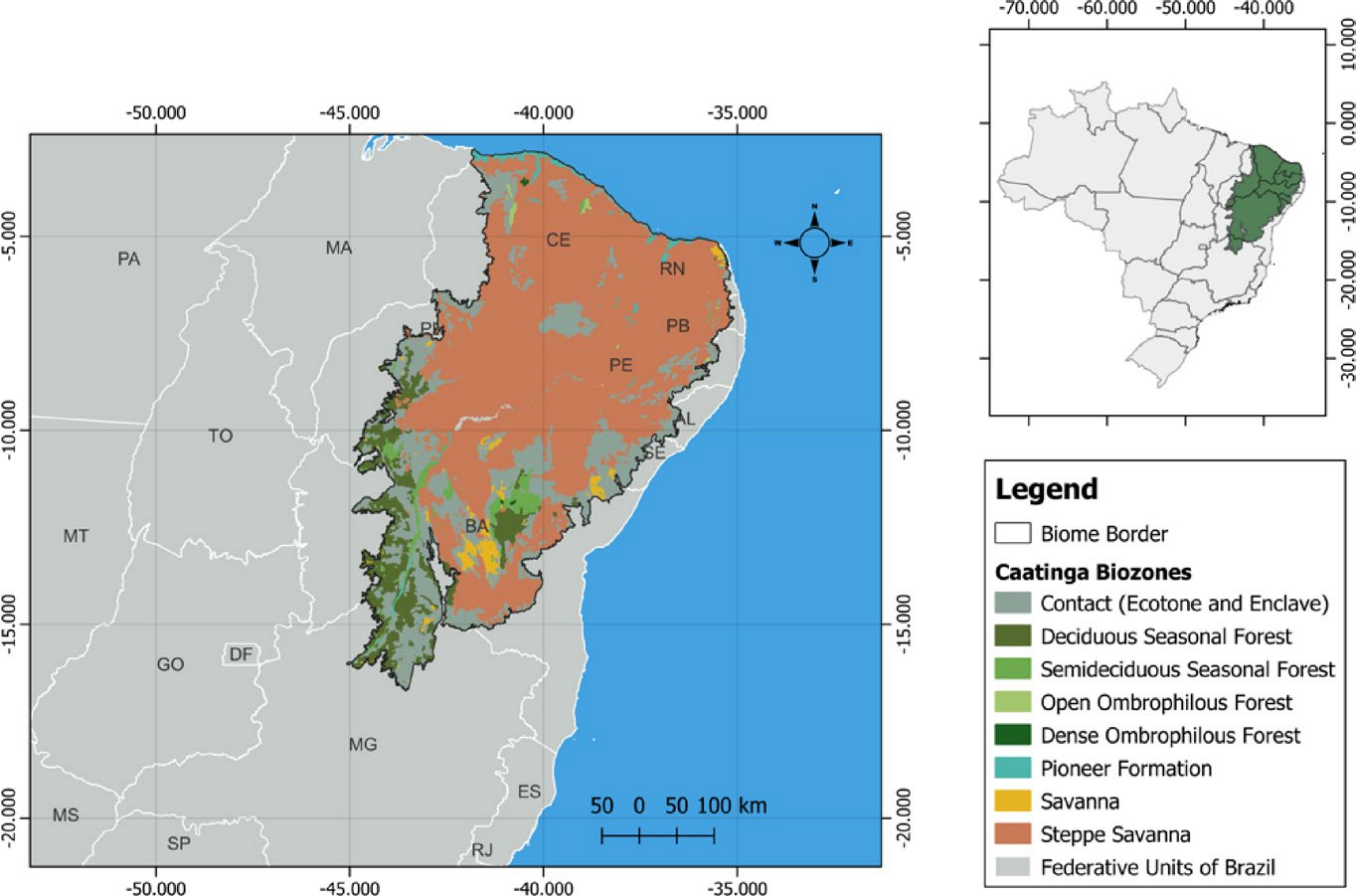
Location of the Caatinga biome and its phytogeographic biozones. Map showing the spatial extent of the Caatinga biome in northeastern Brazil, highlighting the different phytogeographic subzones (phytoecological regions) that characterize its internal heterogeneity. Boundaries are based on official biome delimitations.

The biome selected for this study is the Caatinga (Fig. 1), located in the northeastern region of Brazil. It covers approximately 850,000 km^2^ and spans across nine Brazilian states, accounting for about 10% of the national territory^31^. The Caatinga is often described as a dry tropical forest dominated by shrubs and herbaceous plants, with a significant number of endemic species^32^. The prevailing climate is semi-arid, characterized by a prolonged dry season that can last from 5 to 11 months and an average annual precipitation of about 1000 mm^33^. Temperatures in the region are generally high throughout the year, with annual averages ranging from 24 °C to 28 °C. During the hottest months, maximum temperatures can exceed 40 °C, while the thermal amplitude between day and night is also significant due to low humidity levels^33^.

The soils of the Caatinga form a complex mosaic that supports the development of diverse vegetation adapted to these conditions. In general, the soil classes found in this biome are shallow, rocky, and of low fertility^34^. Approximately 70% of the Caatinga terrain is of crystalline origin (hard bedrock that hinders water storage), while the remaining 30% is associated with sedimentary terrains that are more favorable to water retention^35^. As a result, due to the variability of soils and topography, the Caatinga exhibits a heterogeneous landscape.

### Orbiting carbon observatory

The Orbiting Carbon Observatory-2 (OCO-2) was developed by NASA to remotely monitor atmospheric CO_2_ on a global scale^13^. OCO-2 measures absorption in the oxygen A-band near the 0.76 μm wavelength and in the strong and weak CO_2_ bands at 1.6 μm and 2.6 μm, respectively^11,36^. These parameters are essential for the proper functioning of the ACOS (Atmospheric CO_2_ Observations from Space) retrieval algorithm, which is used to estimate the column-averaged atmospheric CO_2_ concentration (XCO_2_)^36–38^.

Additionally, the sensors onboard the OCO-2 mission provide a spatial resolution of less than 4 km^2^, with each measurement frame containing up to eight individual sampling footprints^39,40^. In this study, we used Level 2, version 10 bias-corrected Lite Files Full Physics data for OCO-2^41^, considering all observation modes: Nadir, Glint, and Target. We included observations that passed cloud screening (quality_flag = 0), as well as those of lower quality (quality_flag = 1) collected between 2015 and 2023 across the entire Brazilian territory. These years were selected because the mission was already operational and providing year-round observations up to the time this study was conducted (January 2025). The OCO-2 Level-2 soundings used in this study represent individual retrievals with an effective revisit frequency of approximately 16 days. To support the spatial-temporal analysis performed here, all valid L2 soundings were aggregated into monthly means for each Caatinga biozone, providing a consistent temporal resolution suitable for detecting seasonal and interannual anomalies in XCO_2_.

### Anomaly model

The XCO_2_ anomaly model was calculated as described by Hakkarainen et al.^13^, where positive anomaly values are interpreted as carbon sources and negative values (below zero) as carbon sinks. These two characteristics are associated with anthropogenic activities, such as fossil fuel combustion, as well as biospheric processes, including forest growth and photosynthetic activity.

Accordingly, XCO_2_ anomalies are computed as the difference between the observed XCO_2_ value at a given grid cell and the daily median derived from the distribution of all valid OCO-2 observations acquired globally on the same day. This daily median represents a background XCO_2_ level and is used to highlight relative spatial anomalies, as expressed in Eq. (1):

1 $$\:{XCO}_{2(i,j)}^{anom}={XCO}_{2(i,j)}-Me\left({XCO}_{2\left(j\right)}\right)$$

Where $\:{XCO}_{2(i,j)}$, is the *i-th* observation of XCO_2_ on day *j*, and $\:Me\left({XCO}_{2\left(j\right)}\right)$ median $\:\left({XCO}_{2\left(j\right)}\right)$ value for day *j*.

This model removes the background component of CO_2_ concentration, reducing biases caused by spatial variations and regional patterns in the data. This approach has been widely applied to investigate emission sources associated with biomass burning and fossil fuel use^17,25^, as well as to identify natural signals^14,19^. To explore these natural signals and their relationship with ecosystem functioning, we processed time series of biophysical and climatic variables including the Normalized Difference Vegetation Index (NDVI), Enhanced Vegetation Index (EVI), Land Surface Temperature (LST), and cumulative precipitation using the Google Earth Engine (GEE) platform. GEE enables efficient and scalable access, manipulation, and analysis of large volumes of geospatial data, providing an integrated environment for evaluating vegetation dynamics and climate-carbon interactions across broad spatial and temporal scales^42^.

To ensure consistency before computing correlations, all datasets with different native resolutions were spatially and temporally harmonized. XCO_2_ measurements from OCO-2, which present irregular spatial sampling, were aggregated into monthly means for each biozone. MODIS-derived vegetation indices (250 m, 16-day) and LST (1 km, daily) were composited into monthly averages, matching the aggregation period used for XCO_2_ and precipitation. Precipitation data were also summarized monthly and spatially aggregated to the same biozone extent. These harmonization procedures minimize scale-related biases, although we acknowledge that uncertainties may remain due to differences in the original resolutions of each product.

### Vegetation indices (NDVI and EVI)

The NDVI (Eq. 2) and EVI (Eq. 3) indices were obtained from the MODIS MOD13Q1 product, Collection 6.1 (MODIS/061/MOD13Q1), with a spatial resolution of 250 m and a temporal resolution of 16 days. Time series data were collected from January 1, 2015, to December 31, 2022. Both NDVI and EVI were considered in this study because they provide complementary information on vegetation dynamics in dryland ecosystems such as the Caatinga. NDVI is widely used as an indicator of vegetation greenness, but it can saturate under moderate-to-high canopy density and be strongly influenced by soil background reflectance, which is common in semi-arid landscapes. EVI, in turn, incorporates corrections for soil and atmospheric effects, offering improved sensitivity in sparse vegetation conditions. Thus, using both indices allows a more robust assessment of vegetation responses to climatic variability in the region. NDVI is used as an indicator of photosynthetic activity and vegetation density, whereas EVI provides greater sensitivity in areas with dense canopy or high biomass, reducing the saturation effects commonly observed in NDVI^43^.

2 $$\:NDVI\:=\:\frac{NIR\:+\:RED}{NIR-RED}$$

Where: NIR = near-infrared reflectance (MODIS band 2); RED = red reflectance (MODIS band 1)

3 $$\:EVI=G\cdot\:\:\frac{NIR+{C}_{1}\cdot\:RED}{{C}_{2}\cdot\:BLUE+L.NIR-RED}$$

using the coefficients recommended by Huete et al.^44^: *G* = 2.5 (gain factor); *C*_*1*_ = 6.0 (red coefficient); *C*_*2*_ = 7.5 (blue coefficient); *L* = 1.0 (canopy background adjustment factor); *BLUE* = blue reflectance (MODIS band 3).

### Land surface temperature (LST) and precipitation

Land surface temperature data were obtained from the MODIS MOD11A2 product, Collection 6.1 (MODIS/061/MOD11A2), which provides average LST values at a spatial resolution of 1 km and a temporal resolution of 8 days. This variable is useful for analyzing thermal patterns associated with ecological processes such as water stress and fire risk^45^.

4 $$\:LST=\:{T}_{4}+a\left({T}_{4}-{T}_{5}\right)+b{\left({T}_{4}-{T}_{5}\right)}^{2}+c\left(1-\epsilon\:\right)+d\left(\varDelta\:\epsilon\:\right)$$

Where: *T*_*4*_ and *T*_*5*_: Brightness temperatures measured in thermal infrared bands 4 and 5 (typically centered at 11 μm and 12 μm, respectively); *a*,* b*,* c*,* d* are empirical coefficients determined through calibration based on atmospheric and surface data; *ε*: Mean surface emissivity.

$\:\varDelta\:\epsilon\:={\epsilon\:}_{4}-{\epsilon\:}_{5}$ Difference in emissivity between the two thermal bands.

Monthly precipitation data were obtained from the TerraClimate database, available in the *IDAHO_EPSCOR/TERRACLIMATE* collection, with a spatial resolution of ~ 4 km. The time series covers the period from 2015 to 2022, encompassing both seasonal and interannual events. This database combines observational data with climate reanalyses and is widely used in studies of environmental dynamics and climate change at regional scales^46^.

### Statistical analyses

The statistical analyses aimed to identify patterns, trends, and relationships between atmospheric CO_2_ concentration (XCO_2_) and biophysical and climatic variables across the Brazilian territory from 2015 to 2023.

Initially, the Local Moran’s I index (LISA) was applied to detect significant spatial clusters of XCO_2_ anomalies within the Caatinga biome, considering its biozones. This allowed the identification of hotspots (areas with high anomalous CO_2_ concentrations) and coldspots (areas with low concentrations), enabling a regionalized analysis of carbon sources and sinks and their relation to land use and cover.

Subsequently, Pearson correlation analyses were conducted between XCO_2_ anomalies and environmental variables such as precipitation, LST, NDVI, and EVI. To address the limitations of Pearson correlations in the presence of multicollinearity, partial correlation analyses were further performed to isolate the independent relationship between XCO_2_ and each predictor. Additionally, multicollinearity was diagnosed using the Variance Inflation Factor (VIF), which indicated severe redundancy between NDVI and EVI (VIF > 36), while the remaining variables remained within acceptable thresholds. These procedures ensured more robust inference regarding the dominant environmental predictors of XCO_2_ variability.

Additionally, Pearson correlation analyses were conducted between XCO_2_ anomalies and environmental variables such as precipitation, LST, NDVI, and EVI. Statistically significant correlations (t-test, *p* < 0.05) allowed the inference of possible associations between carbon fluxes and biophysical factors such as photosynthetic activity, water stress, and rainfall seasonality.

To assess the normality of residuals and homogeneity of variances (homoscedasticity) in the statistical models, the Shapiro–Wilk test and the Levene test were applied at a significance level of 1% (*p* > 0.01). Testing for normality and homoscedasticity was essential to validate the assumptions of regression analyses and subsequent mean comparisons. To evaluate statistically significant differences between distinct groups (e.g., regions, seasonal periods, or climatic regimes), an analysis of variance (ANOVA) based on the F-test was performed at a 5% significance level.

Furthermore, to evaluate trends and seasonal patterns throughout the time series of XCO_2_ anomalies, the ARIMA (AutoRegressive Integrated Moving Average) model was employed. This model enabled the detection of cyclical behaviors and temporal persistence in the data, assisting in identifying structural or continuous changes in atmospheric CO_2_ dynamics. Prior to modeling, all-time series were subjected to the Augmented Dickey–Fuller (ADF) test to assess stationarity. When necessary, differencing (integration) or the inclusion of seasonal terms was applied to the models, based on the Akaike Information Criterion (AIC) and residual diagnostics. All statistical analyses and modeling were performed using R software (R Core Team, 2025)^47^.

In the ARIMA modeling step, three model structures were evaluated for each bioclimatic zone: ARIMA (0,1,0), ARIMA (1,1,0), and ARIMA (1,1,2). Model selection was based on the lowest Akaike Information Criterion (AIC) and diagnostic checking of residuals (Shapiro–Wilk and Ljung–Box tests). The prediction intervals displayed in Fig. 5 (colored shaded areas) correspond to the forecast horizon generated by the best-fit ARIMA model for each bioclimatic zone, and do not represent observational uncertainty. All forecasts were produced for a 12-month projection beyond the observed period.

## Results

### Temporal and seasonal variability of XCO_2_ anomalies

The intra-annual analysis of monthly XCO_2_ anomalies throughout the time series reveals a consistent cyclic pattern, characteristic of the strong seasonality of the Caatinga biome. In all evaluated years, positive anomaly peaks are observed between January and March, corresponding to the late dry season and the onset of the rainy period, when photosynthetic activity remains reduced and CO_2_ uptake is not yet fully reestablished. As the rainy season progresses, between April and September, there is a systematic decrease in the anomalies, with values close to zero or negative in most years.

**Fig. 2:**
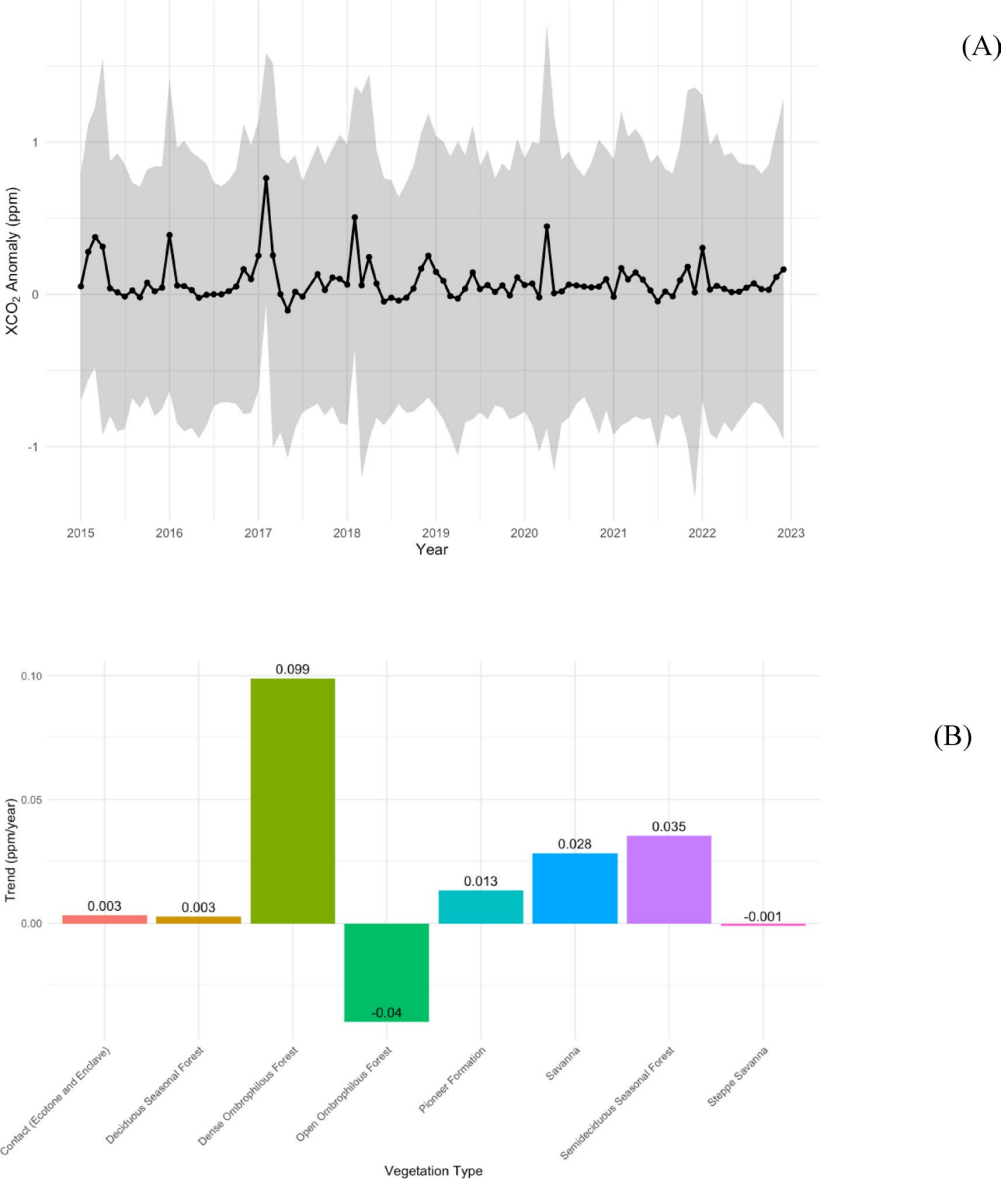
(**A**) Interannual trends of XCO_2_ anomalies by vegetation type, where the solid line represents the median XCO_2_ anomaly and the shaded area indicates the variability range of anomalies over time (reflecting the dispersion of positive and negative values). (**B**) Seasonal cycle of monthly XCO_2_ anomalies across the study period.

Although the annual cycle is recurrent, the amplitude of this oscillation exhibits interannual variation, indicating differences in ecosystem behavior over time. This is evident in years such as 2015 and 2017, which show more pronounced seasonal peaks, whereas 2019 and 2021 display a more attenuated seasonal variation (Fig. 2a). Additionally, in some years, a delay in the transition to negative anomalies is observed, with positive values extending into April, reflecting shifts in the timing of vegetation recovery among different years.

The temporal trends of XCO_2_ anomalies differed among vegetation types (Fig. 2b). *Dense Ombrophilous Forest* showed the largest positive trend, with XCO_2_ anomalies increasing at 0.099 ± 0.020 ppm per year (*p* < 0.001), indicating a consistent upward trend over the study period. *Semideciduous Seasonal Forest* (0.035 ± 0.005 ppm yr^−1^, *p* < 0.001), *Savanna* (0.028 ± 0.006 ppm yr^−1^, *p* < 0.001), *Pioneer Formation* (0.013 ± 0.006 ppm yr^−1^, *p* = 0.031), and *Contact (Ecotone and Enclave)* (0.0033 ± 0.0013 ppm yr^−1^, *p* = 0.013) also exhibited positive trends, although of smaller magnitude.

In contrast, *Open Ombrophilous Forest* exhibited a significant negative trend (−0.040 ± 0.013 ppm yr^−1^, *p* = 0.002), suggesting a gradual decrease in XCO_2_ anomalies. *Deciduous Seasonal Forest* (0.0027 ± 0.0020 ppm yr^−1^, *p* = 0.171) and *Steppe Savanna* (−0.00095 ± 0.00073 ppm yr^−1^, *p* = 0.190) did not show statistically significant trends. These results highlight that the temporal evolution of XCO_2_ anomalies is strongly dependent on vegetation type, with some biomes acting as increasing sources or sinks over the analyzed period.

### Spatial and temporal distribution of XCO_2_ anomalies (2015–2023)

**Fig. 3:**
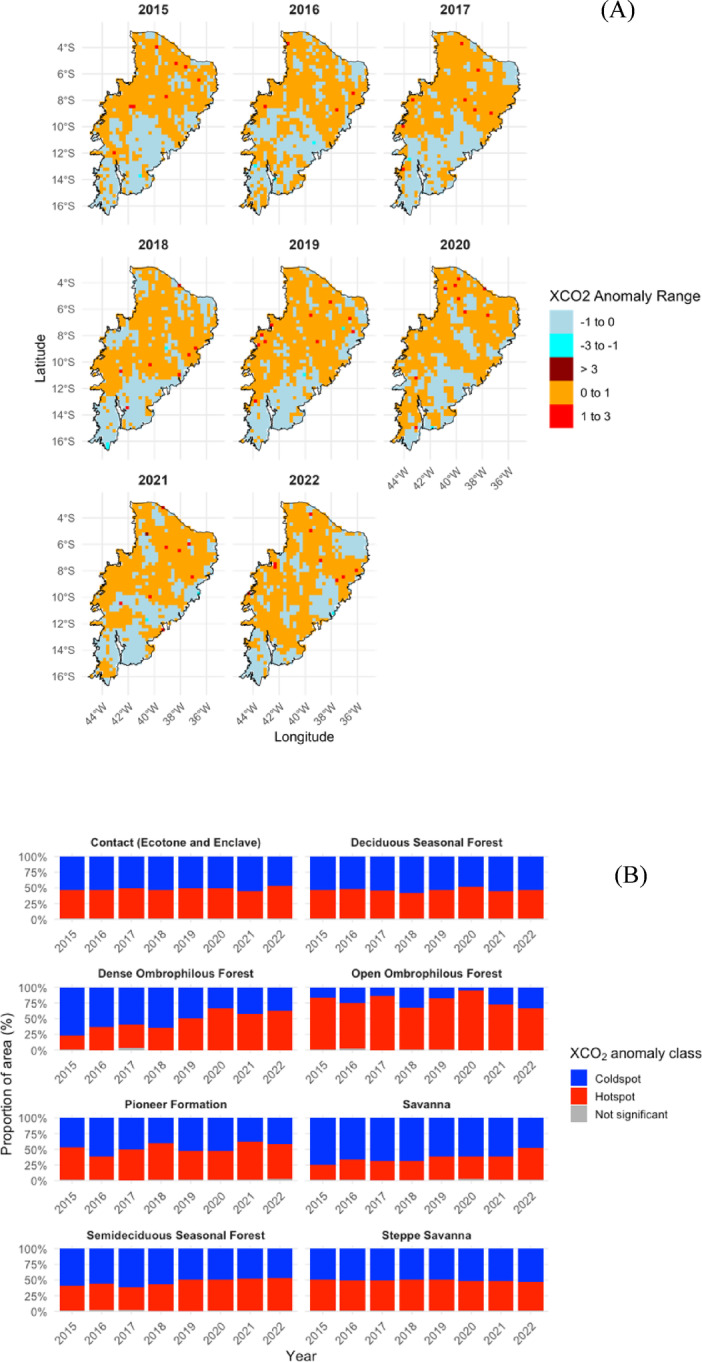
(**a**) Spatial distribution of XCO_2_ anomalies (ppm) across the Caatinga biome between 2015 and 2023, derived from OCO-2 satellite observations. The maps were produced by interpolating monthly XCO_2_ anomalies and are presented by phytophysiognomic biozones in a faceted layout. Negative anomalies (blue shades) indicate carbon sink behavior, whereas positive anomalies (red shades) represent potential CO_2_ emission sources, highlighting spatial heterogeneity among vegetation types and the occurrence of localized hotspots linked to climatic and anthropogenic drivers. (**b**) Interannual variation in the proportional area (%) of the Caatinga biome classified by XCO_2_ anomaly classes over the same period, summarizing the relative contribution of sink- and source-dominated conditions across years.

The spatial distribution of XCO_2_ anomalies across the Caatinga biome between 2015 and 2023 (Fig. 3a) reveals recurrent spatial patterns accompanied by marked interannual variability. Quantitatively, negative XCO_2_ anomalies consistently dominated the biome, accounting for approximately 55–70% of the total area in most years (Fig. 3b), particularly between 2015 and 2017, when moderate negative anomalies were spatially widespread across central and southern regions.

From 2018 onward, the proportion of area exhibiting positive anomalies increased, reaching up to 30–35% of the biome in 2019 and 2020 (Fig. 3b). These years also showed the emergence of spatially clustered hotspots in the northern and northeastern portions of the Caatinga (Fig. 3a), suggesting enhanced CO_2_ source behavior potentially linked to land-use change, wildfire activity, or prolonged water stress.

In 2021 and 2022, a partial recovery toward sink-dominated conditions was observed, with negative anomalies again representing approximately 60% of the biome area, particularly in the southern sector (Fig. 3b). Nevertheless, a persistent fraction of 10–20% of the area continued to exhibit positive anomalies, highlighting the heterogeneous response of the Caatinga to climatic and environmental pressures.

### Spatial patterns of local XCO_2_ autocorrelation across Caatinga biozones

**Fig. 4:**
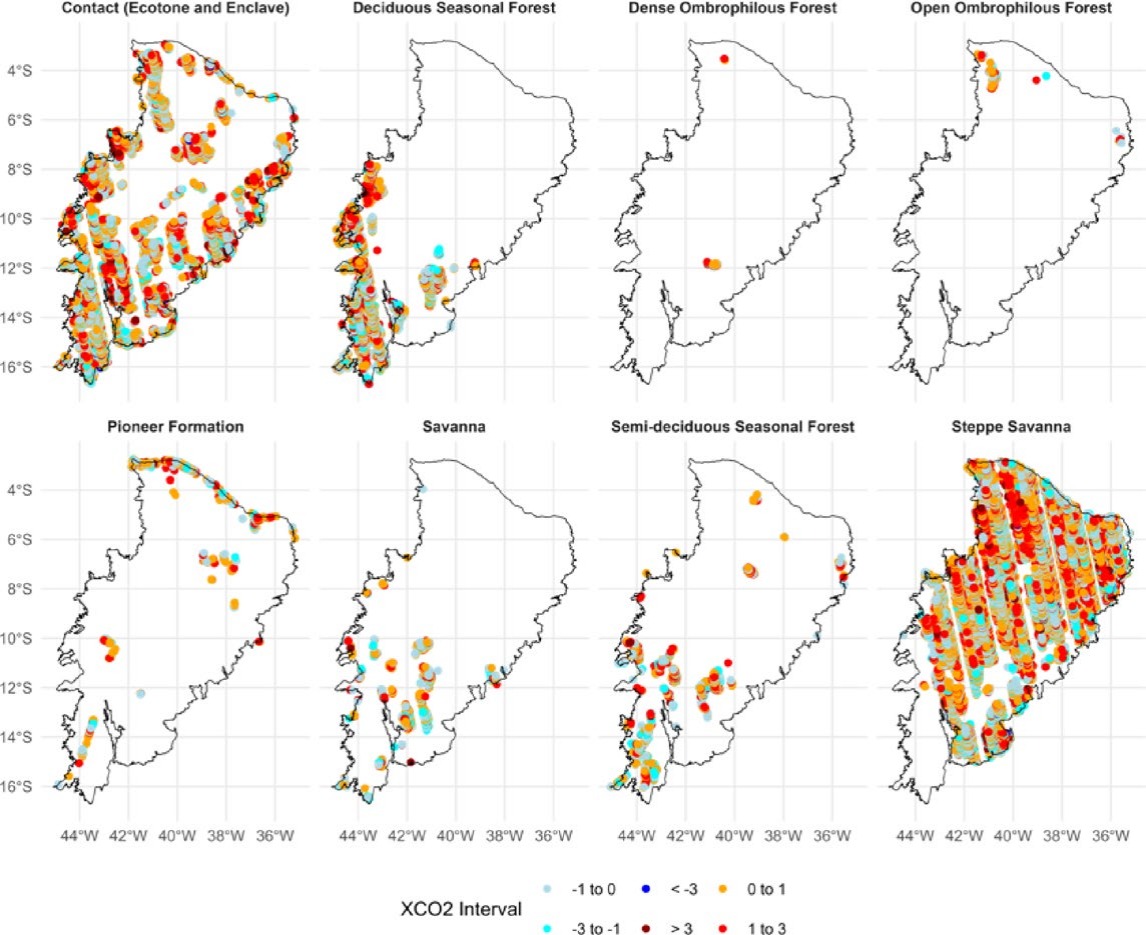
Local spatial autocorrelation (LISA – Local Indicators of Spatial Association) of XCO_2_ anomalies in the Caatinga biome between 2015 and 2023. Clusters classified as high–high (red) indicate areas with consistently elevated anomalies, while low–low clusters (blue) represent persistent sinks. Outliers are shown as high–low (light red) and low–high (light blue), capturing local contrasts in carbon flux dynamics. The results highlight spatial dependence and heterogeneity among phytophysiognomic biozones, reflecting the combined influence of ecological characteristics and anthropogenic pressures.

The spatial analysis of XCO_2_ anomalies based on the local Moran’s I index (Fig. 4) revealed distinct autocorrelation patterns within the various phytophysiognomic biozones of the Caatinga. The biozones exhibited significant variability in the distribution of positive spatial clusters (high-high), negative clusters (low-low), and spatial outliers (high-low and low-high), underscoring the complexity of CO_2_ emission and absorption dynamics within the biome.

The *Steppe-Savanna* biozone, which covers a broad area in the southeastern Caatinga, presented the largest and most significant high-high clusters, particularly concentrated in the central-eastern portion (Fig. 4). In contrast, zones such as the *Seasonal Deciduous Forest* and *Contact areas* exhibited a more mixed distribution, with the simultaneous occurrence of both positive and negative clusters.

Smaller biozones, such as the *Pioneer Formation* and the *Dense Ombrophilous Forest*, showed a lower density of significant anomaly points. This may reflect both data resolution limitations and the lower spatial variability in XCO_2_ dynamics in these regions. Nonetheless, isolated points of both positive and negative autocorrelation were observed, indicating that even in these areas, hotspots and coldspots of CO_2_ emission and absorption may occur (Fig. 4).

**Fig. 5:**
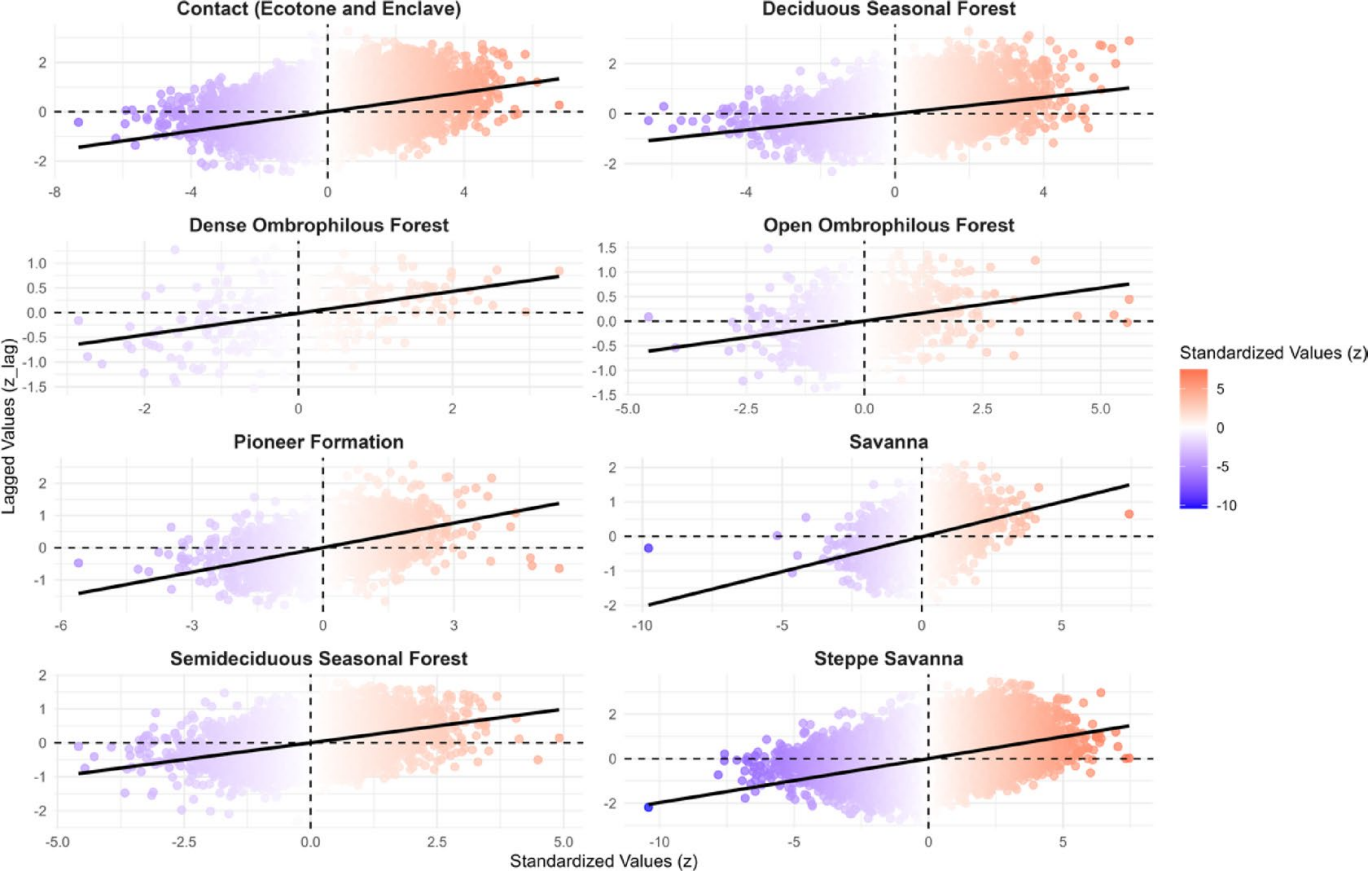
Moran scatterplots of XCO_2_ anomalies by Caatinga phytophysiognomic biozones from 2015 to 2023. Each plot shows the relationship between standardized XCO_2_ anomalies (x-axis) and their spatial lag (y-axis). The regression line indicates the slope corresponding to the local Moran’s I value, reflecting the degree of spatial autocorrelation within each vegetation type. Steeper slopes, as observed in the Steppe Savanna and Contact areas, indicate stronger spatial structure and clustering, while flatter slopes (e.g., Dense Ombrophilous Forest) suggest weaker spatial dependence.

The local Moran’s index scatterplots (Fig. 5) reinforce these observations. All biozones displayed a general tendency toward positive spatial correlations between XCO_2_ values and their neighboring values, indicating significant spatial dependence. The regression slope varied among biozones, with a steeper inclination observed in the *Steppe-Savanna* (slope = 0.58), confirming a higher degree of spatial clustering in that domain. The slope values ranged from 0.09 in *Dense Ombrophilous Forest* to 0.58 in *Steppe-Savanna*, indicating variability in the strength of spatial association across vegetation types. The slopes for each vegetation type are shown in the respective panels.

### Analysis of XCO_2_ anomalies by vegetation type with ARIMA forecasts

**Fig. 6:**
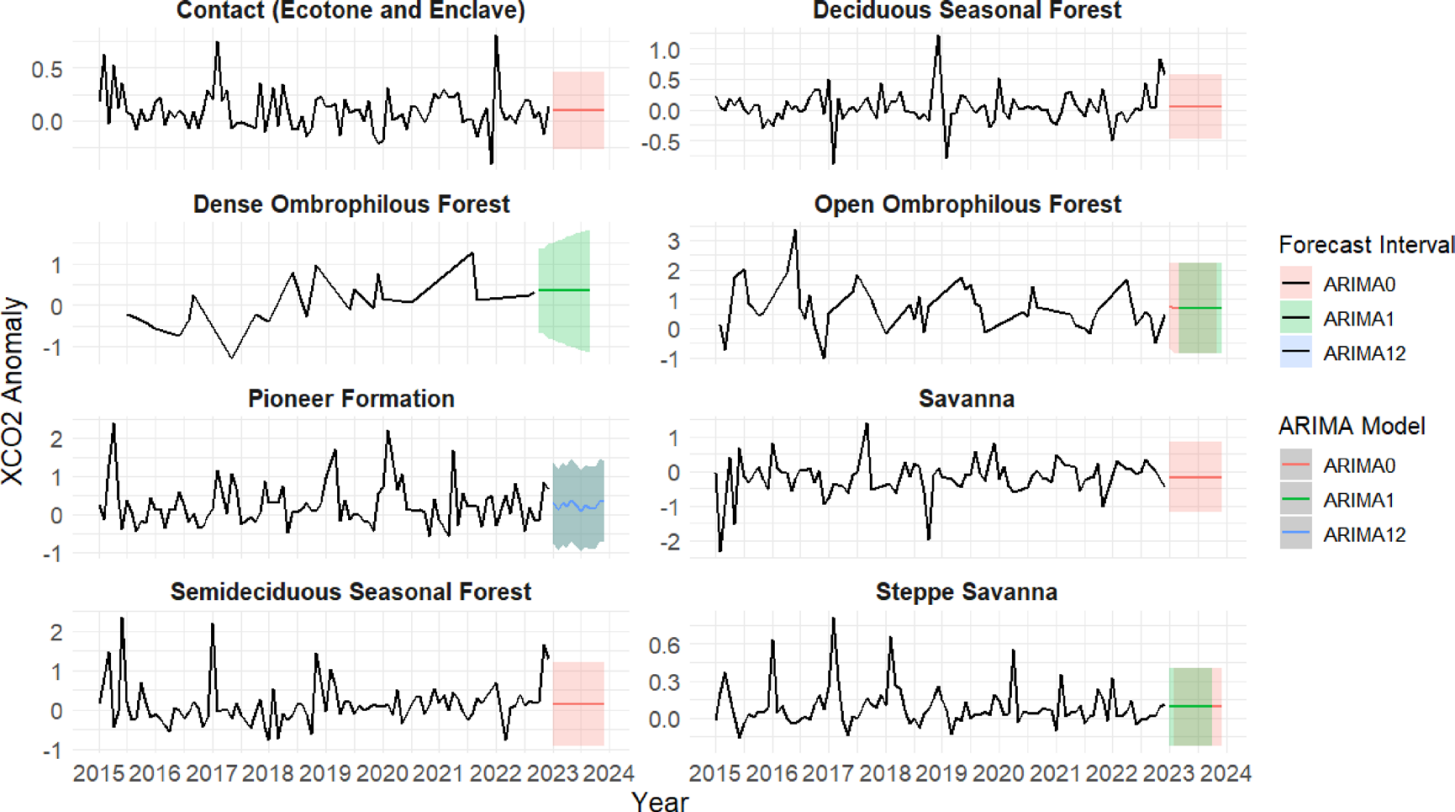
Temporal dynamics of XCO_2_ anomalies in Caatinga phytophysiognomic biozones from 2015 to 2023 with ARIMA model forecasts. Solid lines represent observed anomalies, while shaded areas denote fitted ARIMA predictions and confidence intervals. Distinct model structures (e.g., ARIMA (0,0,0), ARIMA (1,0,0), ARIMA (1,1,1)) highlight the contrasting carbon dynamics among vegetation types, with stationary regimes in savanna formations, seasonal patterns in pioneer vegetation, and upward trends in ombrophilous forests.

Figure 6 presents the time series of XCO_2_ anomalies for the different biozones of the Caatinga from 2015 to 2023, together with forecasts generated using ARIMA models. The temporal dynamics of the anomalies differ considerably across the biozones, reflecting distinct ecological regimes. These variations suggest that each biozone exhibits a unique pattern of atmospheric CO_2_ fluctuation, potentially associated with differences in emission and removal processes over time.

The time series for the *Contact areas* biozone (ecotone and enclave) was modeled with an ARIMA (0,0,0) with a non-zero mean, indicating no trend or significant autoregressive structure, but persistence of positive anomalies, especially between 2021 and 2022. The forecasts suggest the continuation of this pattern in the short term. A similar pattern was observed in the *Deciduous Seasonal Forest biozone*, whose series showed moderate variations and episodes of increasing anomalies in recent years. The ARIMA (0,0,0) model with a non-zero mean adjusted to this biozone also indicated a statistically stationary regime around a distinct non-zero mean, reflecting stability, though with signs of recent increase (Fig. 6).

In the *Pioneer Formation* biozone, a well-defined seasonal behavior was observed, with regular peaks that justified the application of an ARIMA (0,0,0) (1,0,0)^12^ model, incorporating an annual seasonal component (Fig. 6). This pattern suggests that variations in XCO_2_ anomalies in this biozone are strongly associated with seasonal cycles, possibly related to vegetation phenology or the alternation between growth and senescence periods. In the case of the *Open Ombrophilous Forest* biozone, the series exhibited significant oscillations and an upward trend in recent years. The most appropriate model was ARIMA (0,0,1) with a zero mean, which captured short-term dependencies and low-order fluctuations, indicating a possible increase in anomalies. The *Dense Ombrophilous Forest* biozone, in turn, showed a growing trend between 2019 and 2022, though with smoother fluctuations. The best fit for this biozone was the ARIMA (1,1,1) model with a non-zero mean, evidencing that the series responds to past shocks with short-term memory, but with a consistent trend of increasing anomalies.

The *Dense Ombrophilous Forest* class exhibited behavior marked by sharp peaks and abrupt variations over time. This pattern was best fitted by the ARIMA (1,1,1) model, which combines differencing, autoregressive, and moving average components being the most complex among the applied models (Fig. 6). This adjustment indicates that the series has a long memory and requires transformations to achieve stationarity, possibly reflecting intense seasonal disturbances such as caused by fires or recent deforestation. For the *Savanna* biozone, stationary behavior was observed around a slightly negative mean until 2021, followed by stability in subsequent years. The ARIMA (0,0,0) model with a non-zero mean was considered sufficient to represent the series, indicating no significant trends or dependence structures. Finally, the *Steppe-Savanna* biozone stood out for presenting a marked seasonal pattern, with recurring annual peaks. In this case, an ARIMA (1,0,0) model with a non-zero mean was adjusted, whose projection indicates the persistence of seasonal cycles and a slight upward trend in anomalies in future periods.

### Comparison of environmental covariates among vegetation types

**Fig. 7:**
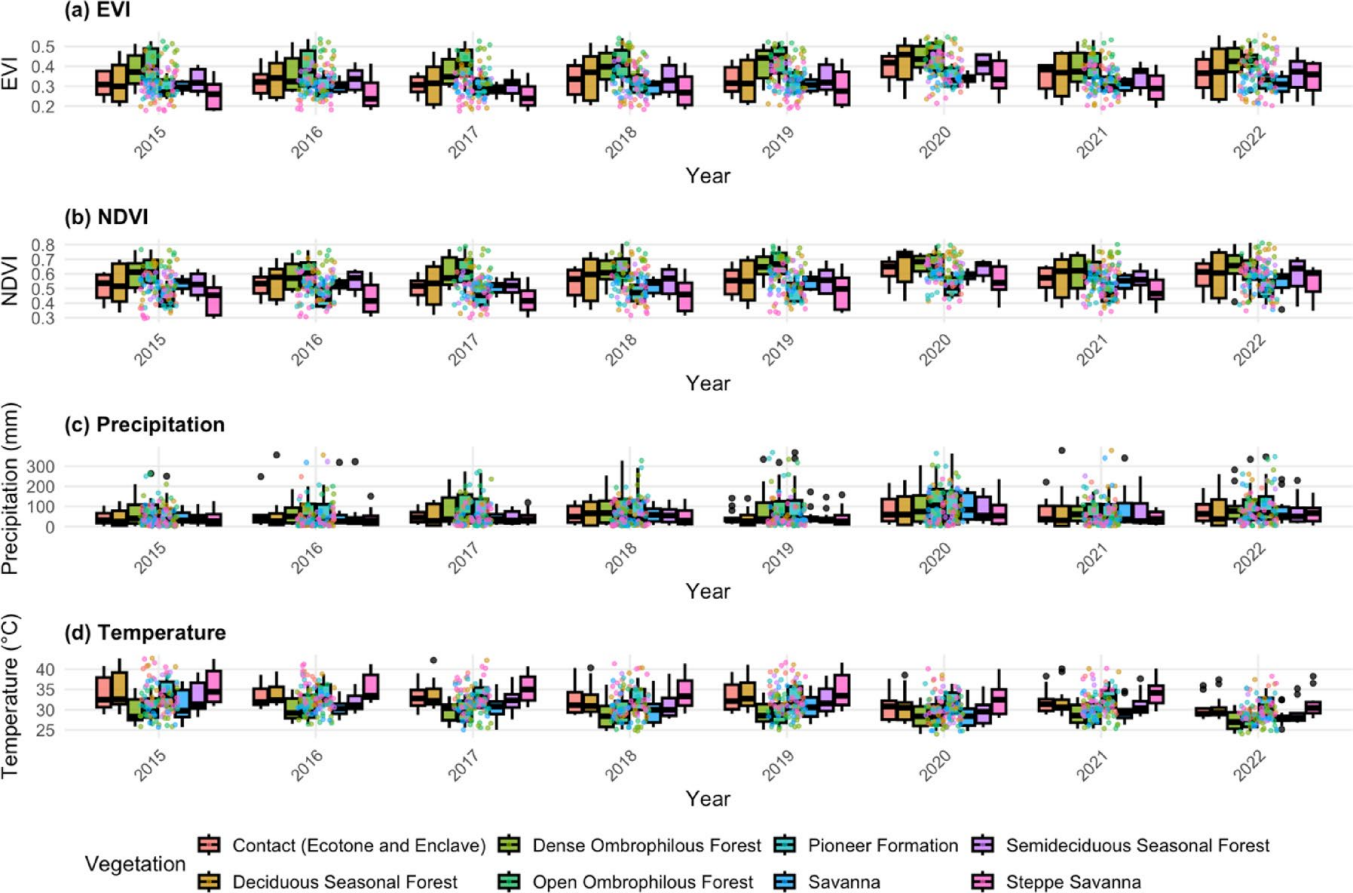
Climatic variability across Caatinga phytophysiognomic biozones. Results of ANOVA and Tukey’s post hoc test (*p* < 0.05) applied to annual mean temperature (°C) and annual accumulated precipitation (mm) for the period 2015–2023. Boxplots show statistically distinct groups among vegetation types, with higher precipitation in ombrophilous forests and pioneer formations, and higher mean temperatures in savanna and steppe regions.

 For EVI (Fig. 7a), a general pattern similar to that of NDVI was observed, but with more pronounced differentiation between groups. The highest EVI means were found in the *Dense Ombrophilous Forest* and *Ombrophilous Forest*, both statistically superior to the other biozones. On the other hand, *Savanna*, and *Steppe*-*Savanna* had the lowest EVI values, clustering into significantly distinct categories from the forest formations. Thus, EVI due to its higher sensitivity to dense vegetation and lower saturation in areas with high canopy cover more clearly highlighted the structural contrasts among the different biozones.

For NDVI (Fig. 7b), higher values were observed in the *Dense Ombrophilous Forest*, and *Open Ombrophilous Forest* biozones. These biozones differed significantly from the more open formations such as *Steppe*-*Savanna* and *Pioneer Formation* which showed the lowest average NDVI values. These results reflect a greater degree of vegetation cover and biomass density in the forest formations compared to the *Savanna* and *Pioneer formations*, which are more sparse, seasonal, or subject to disturbance.

Although these vegetation structure patterns are well established in the literature, their inclusion here is crucial because they provide the ecological basis supporting our atmospheric findings: biozones with denser canopy cover and higher carbon assimilation capacity are the same areas where we observe stronger negative XCO_2_ anomalies. Thus, NDVI and EVI are key to interpreting how vegetation functional differences modulate carbon dynamics across the Caatinga.

Therefore, the coherence between the two indices reinforces the robustness of the analyses and underscores the relevance of these indicators in monitoring the vegetation structural conditions, especially in the context of carbon dynamics and XCO_2_ anomalies studies.

### Variation in temperature and precipitation among vegetation types

Figure 7c and d presents the results of the analysis of variance (ANOVA), followed by Tukey’s multiple comparison test (*p* < 0.05), applied to the environmental variables of mean precipitation (mm) and temperature (°C), considering the different biozones. Based on the results, it was found that biozones such as *Open Ombrophilous Forest*, and *Pioneer Formation* exhibited the highest average precipitation values, differing statistically from the drier biozones, such as *Steppe-Savanna*, which formed a group with the lowest precipitation values.

Regarding mean temperature, significant differences were also observed among the biozones. The highest temperature values were recorded in the *Steppe-Savanna*, *Savanna*, *Pioneer Formation*, and *Contact* areas (ecotone and enclave), which were statistically distinct from the *Dense Ombrophilous Forest* and *Open Ombrophilous Forest* biozones. These latter zones exhibited the lowest average temperatures, highlighting the influence of vegetation structure and canopy density on local microclimatic conditions.

### Independent effects of vegetation and climate drivers on XCO_2_ anomalies

Partial correlation analysis revealed that vegetation activity is the primary independent driver of XCO_2_ anomalies (Table 1). NDVI showed a strong negative association with XCO_2_ (*r* = − 0.625, *p* < 0.05), indicating enhanced biogenic uptake during periods of higher vegetation greenness. EVI also presented a significant correlation (*r* = 0.593, *p* < 0.05), but with an inverted signal compared to the Pearson coefficient, reflecting strong redundancy between vegetation indices. Conversely, temperature and precipitation exhibited weak and non-significant partial correlations (*r* = 0.187 and *r* = − 0.180, respectively; *p* > 0.05), suggesting limited independent influence on XCO_2_ anomalies after accounting for vegetation–climate interactions. Variance Inflation Factor (VIF) values confirmed severe multicollinearity for NDVI and EVI (VIF > 36), while the other predictors remained within acceptable thresholds.

**Table 1:** Artial correlations and multicollinearity diagnostics for environmental predictors of XCO_2_ anomalies.

Response variable	Predictor	Partial *r*	*p*-value	VIF	Multicollinearity
XCO_2_	NDVI	−0.625	< 0.05	39.62	Extreme
XCO_2_	EVI	0.593	< 0.05	36.42	Extreme
XCO_2_	Temp	0.187	> 0.05	3.59	Acceptable
XCO_2_	Precip	−0.180	> 0.05	2.55	Acceptable
XCO_2_	Year	0.085	> 0.05	1.14	None

## Discussion

### Intra-annual variability of XCO_2_ anomalies in the Brazilian Caatinga biome

The seasonal patterns observed in XCO_2_ anomalies reflect a behavior consistent with the phenology of the Caatinga biome. Enhanced biogenic CO_2_ assimilation occurs in response to leaf regrowth and increased foliage during the wet season. With the onset of rainfall and higher soil moisture, vegetation resumes growth, expands leaf area, and intensifies photosynthesis, resulting in greater CO_2_ uptake. This explains the lower (or negative) anomaly values observed in the wetter months^48^. Such patterns are supported by eddy-covariance and remote sensing studies, which show that gross primary productivity (GPP) peaks during the rainy season alongside increases in vegetation indices (e.g., NDVI, EVI), demonstrating the strong link between foliar phenology and photosynthetic capacity^48^. Consequently, our results reinforce that the Caatinga functions as a net carbon sink during the wet months, when biogenic assimilation outweighs respiration and soil emissions^49^.

Conversely, the interannual variability in the amplitude of XCO_2_ anomalies years with pronounced positive peaks versus years with more attenuated seasonality highlights spatial and temporal heterogeneity in ecosystem responses. This heterogeneity is likely driven by variability in rainfall intensity, timing, and distribution, as well as edaphic conditions, vegetation structure, and land-use history. These observations align with prior studies indicating that carbon flux dynamics in the Caatinga are strongly influenced by rainfall seasonality and local environmental factors^49^. Our seasonal cycle analysis further reveals clear differences in temporal trends among vegetation types: Dense Ombrophilous Forest exhibited an increase of ~ 0.099 ppm yr^−1^, whereas Open Ombrophilous Forest showed a negative trend of ~−0.040 ppm yr^−1^. Such results indicate that even within the same biogeographic domain, vegetation cover responds differently to the drivers of atmospheric CO_2_, likely reflecting differences in phenology, productivity, leaf area, and carbon sequestration capacity. Denser vegetation communities appear to retain or accumulate CO_2_ or at least reduce relative emissions more effectively than more open formations, a pattern consistent with evidence that terrestrial carbon sinks in tropical biomes are increasingly spatially heterogeneous and uncertain^50^.

Overall, these findings support the premise that climatic controls, particularly hydrological regimes and rainfall timing, play a decisive role in shaping the carbon–vegetation cycle in the Caatinga. Phenology, modulated by rainfall, directly drives the XCO_2_ anomaly signals, emphasizing the importance of capturing intra-annual variability to accurately assess the biome’s carbon balance. Furthermore, our results have important implications for carbon quantification and biogeochemical modeling in dry biomes: relying solely on annual means or raw atmospheric CO_2_ data without accounting for seasonality and phenology may under- or overestimate the Caatinga’s carbon sequestration potential. Using monthly anomalies, as in this study, provides a robust approach to capture the pulses of carbon assimilation and emission associated with phenology, climate, and hydrological regimes.

### Patterns of XCO_2_ anomalies in the Caatinga (2015–2023)

The predominance of negative and neutral XCO_2_ anomalies (Fig. 2) across much of the Caatinga biome is consistent with its characterization as an efficient carbon sink. Mendes et al.^51^ demonstrated this behavior even under extreme drought conditions, reporting an annual mean flux of − 1.86 µmol m^−2^ s^−1^ during wet years and − 0.81 µmol m^−2^ s^−1^ during severe droughts. This pattern is further supported by findings indicating that the Caatinga ranks among the most effective carbon-sequestering biomes in Brazil, accounting for approximately 45–60% of the CO_2_ absorbed nationwide^52^. These results are also in agreement with previous assessments summarized in Table 2, which collectively highlight the biome’s strong tendency toward net carbon uptake despite environmental variability.

**Table 2:** Summary of the methods employed in the analysis of emissions and environmental variables.

Reference	Location	Method/Variable	Reported value	Conclusion
Mendes et al.^53^	ESEC-Seridó (RN), Brazil	Eddy covariance – NEE, GPP, Rₑco (2014–2015)	NEE: −1.69 and − 1.45 Mg C ha^−1^ yr^−1^	The Caatinga acted as a carbon sink during the studied years.
Schulz et al.^44^	Caatinga regions	Soil sampling – SOC stocks by depth	SOC ≈ 16.9 Mg C ha^−1^ (varies with depth and management)	Grazing reduces soil carbon stocks, especially near the surface.
Silva et al.^54^	Brazilian semiarid (Caatinga)	SOC change under natural regeneration	Increased SOC, N and P in abandoned pastures after regeneration	Natural regeneration helps recover soil carbon and nutrients.
Freitas et al.^55^	Transition zone between Caatinga and Cerrado	Soil carbon and nitrogen stocks in agrosilvopastoral systems	ASP systems maintain or improve SOC and N stocks	Sustainable land-use systems can mitigate soil carbon losses.
Viana-Lima et al.^56^	Caatinga degraded areas	Soil health indicators and SOC under overgrazing vs. restoration	Loss of ~ 14.7 Mg C ha^−1^ in topsoil under overgrazing	Overgrazing strongly degrades soil carbon and ecosystem health.
This study	Caatinga biome (Brazil)	OCO-2 L2 v10 bias-corrected Lite Files; ACOS algorithm; bands at 0.76, 1.6, 2.06–2.10 μm; Nadir/Glint/Target modes; quality_flag = 0 and 1; period 2015–2023	Predominantly positive XCO_2_ anomalies; heterogeneous patterns; emission hotspots; instability in some phytophysiognomies	XCO_2_ anomalies reveal high sensitivity to climate and anthropogenic pressure; sink potential compromised; emission hotspots indicate rising vulnerability; urgent monitoring, protection and restoration needed in the Caatinga

The intensification of negative anomalies between 2015 and 2017, especially in the central-southern and southwestern regions, reflects the strong seasonal control of vegetation activity in the Caatinga. At the onset of the rainy season, vegetation rapidly resumes photosynthesis after prolonged drought, leading to an abrupt increase in CO_2_ uptake and consequently more pronounced negative XCO_2_ anomalies. This behavior is consistent with eddy covariance studies reporting enhanced gross primary productivity (GPP) and ecosystem respiration (Reco) following the first rains, reinforcing the biome’s temporary role as a carbon sink during this transition period^32,57^.

The emergence of intense localized positive XCO_2_ anomalies in 2019 and 2020 supports previous findings linking extreme droughts and fire events to temporary CO_2_ emission peaks^27,34^, potentially reversing the Caatinga typical carbon sink behavior. This interpretation is consistent with the observations of Sayedi et al.^49^, who reported pronounced interannual variability in CO_2_ fluxes within Caatinga ecosystems, driven largely by reductions in gross primary productivity (GPP) and enhanced soil respiration during drought years. These episodic shifts, also documented for other tropical seasonal biomes, reflect the biome’s sensitivity to hydroclimatic extremes and biomass loss, reinforcing the idea that extreme droughts and fire disturbances can temporarily weaken or even invert the region’s carbon uptake capacity^49^. Such dynamics highlight the importance of monitoring short-term anomalies to better understand the resilience and vulnerability of the Caatinga carbon cycle under increasing climate variability.

The partial return to negative anomalies observed in 2021–2022, especially in the southern region, indicates ecosystem resilience and the recovery of carbon removal capacity following the stress events of prior years. Although spatially heterogeneous, this recovery is consistent with dry forest systems that tend to resume sink behavior when water availability is restored^58^. In a global context, the interannual variability observed in the Caatinga resembles that of other tropical biomes, as indicated by OCO-2 satellite data and models such as GONGGA, which report intermittent sink behavior with temporary source pulses during extreme events^59^.

In summary, the spatial and temporal patterns of XCO_2_ anomalies in the Caatinga reflect a combination of climatic drivers (precipitation distribution) and extreme events (droughts, fires), in agreement with the literature that highlights the sensitivity of tropical seasonal forests to water and temperature variability^51^.

### Spatial patterns of local XCO_2_ autocorrelation in Caatinga biozones

The analysis of local spatial autocorrelation patterns of XCO_2_ anomalies across the phytophysiognomic biozones of the Caatinga biome revealed robust evidence of spatial dependence, with the presence of significant clusters (high–high and low–low) and local outliers (high–low and low–high). These results suggest that CO_2_ absorption dynamics in the biome are not random or spatially homogeneous but rather structured in response to regional environmental and anthropogenic drivers.

The predominance of positive clusters (high–high) in the *Steppe-Savanna* biozone, especially in its central–eastern region, indicates areas of potential accumulation or systematic emission of XCO_2_, likely associated with intense anthropogenic pressures such as agricultural expansion, deforestation, and fire use—factors widely documented for this region^60^. This pattern may also reflect intrinsic ecological characteristics of the biozone, including lower vegetation cover density and greater soil exposure, which limit carbon sequestration and favor net emissions.

In contrast, biozones with denser vegetation or mixed ecological structures, such as the *Seasonal Deciduous Forest* and *Contact areas*, exhibited more heterogeneous spatial patterns. The simultaneous presence of positive and negative clusters in these areas may reflect complex interactions among vegetation, soil, and atmosphere, as well as edge effects and transitional ecological conditions. This heterogeneity is consistent with the ecotonal nature of these zones, where small variations in topography, water availability, or land use can lead to significant differences in carbon fluxes^61^.

Smaller biozones, such as *Pioneer Formations* and *Dense Ombrophilous Forest*, showed a lower density of significant local autocorrelation points. This may be due to limitations in the resolution or coverage of satellite-based XCO_2_ measurements, especially in areas with dense vegetation, which can hinder the precision of orbital sensors^36,62,63^. Alternatively, it may indicate lower intrinsic variability in carbon fluxes in these zones, suggesting greater ecological stability or reduced influence from recent disturbances.

The local Moran’s I scatterplots support these findings, showing positive correlations between XCO_2_ values and their neighbors in all biozones analyzed. The steeper slope observed in the Savanna–Steppe biozone indicates a stronger spatial structure in this domain, reinforcing its vulnerability to changes in the carbon biogeochemical cycle. These results are consistent with previous studies reporting increasing environmental degradation in the region, with direct impacts on greenhouse gas dynamics^15,27^.

#### Analysis of XCO_2_ anomalies by vegetation type with ARIMA forecasts

The temporal analysis of XCO_2_ anomalies across the different phytophysiognomic biozones of the Caatinga biome revealed distinct patterns in atmospheric carbon behavior, reflecting ecological and functional contrasts among vegetation types. Modeling with ARIMA (AutoRegressive Integrated Moving Average) captured stationary structures, seasonal dynamics, and long-term trends, offering a quantitative interpretation of XCO_2_ fluctuations from 2015 to 2022.

Biozones characterized by structural stability, such as *Contact areas* (ecotones/enclaves), *Deciduous Seasonal Forest*, and *Savanna*, were adequately modeled with ARIMA (0,0,0) with a non-zero mean. In these cases, the absence of autoregressive and moving-average components indicates stationarity around a constant mean, which nonetheless reflects persistent positive anomalies in recent years potentially linked to sustained anthropogenic pressures or a gradual decline in sequestration capacity^15,63^.

Biozones with pronounced seasonal modulation displayed recurring peaks likely associated with alternating periods of vegetative growth and senescence. The *Pioneer Formation* showed a well-defined annual cycle, best captured by a seasonal ARIMA (0,0,0) (1,0,0)^12^, highlighting the influence of annual climatic forcing. The *Steppe- Savanna* also presented marked seasonal behavior with recurring annual peaks; its dynamics were satisfactorily summarized by an ARIMA (1,0,0) with a non-zero mean, indicating persistence and a slight upward tendency in anomalies, consistent with a seasonally modulated regime^64,65^.

In wetter forested biozones, XCO_2_ anomalies exhibited increasing trends, suggesting recent shifts in carbon dynamics. The *Open Ombrophilous Forest* was best fitted by ARIMA (0,0,1) with a zero mean, capturing short-term dependencies and low-order fluctuations amidst a recent rise in anomalies. The *Dense Ombrophilous Forest* required a more complex ARIMA (1,1,1) with a non-zero mean, indicating response to past shocks with short-term memory and a consistent upward trend since ~ 2019, alongside occasional sharp oscillations that may reflect disturbances such as fire or edge effects from land-use change^36,60^.

Overall, these results demonstrate that the different Caatinga vegetation types respond in distinct ways to climatic and environmental controls most notably rainfall seasonality, soil moisture dynamics, canopy structure, and phenological strategies. Preserved areas and mature forest formations tend to exhibit more stable and predominantly negative XCO_2_ anomalies, reflecting stronger carbon uptake associated with deeper root systems, higher biomass, and greater functional resilience. In contrast, remnant fragments, open shrublands, and anthropogenically impacted zones show more variable and often positive anomalies, driven by heightened sensitivity to hydrological stress, reduced canopy cover, and the influence of land-use activities. Together, these patterns highlight how the interplay between ecological integrity and climatic forcing shapes the spatial heterogeneity of atmospheric CO_2_ behavior across the Caatinga biome^66^. The diversity of fitted models reinforces the need for spatially and ecologically differentiated monitoring of carbon fluxes across the biome. The coexistence of increasing trends and seasonal patterns in several biozones points to heightened vulnerability to climate change and land-use pressures, underscoring the importance of evidence-based mitigation strategies^15,67^.

### Influence of climatic variables in Caatinga biozones: temperature and precipitation

Figure 7 shows the ANOVA results, complemented by Tukey’s multiple comparisons test (*p* < 0.05), applied to annual mean temperature (°C) and annual accumulated precipitation (mm) across Caatinga biozones. The tests indicate statistically significant climatic differences among vegetation types.

In terms of precipitation, the *Open Ombrophilous Forest* and *Pioneer Formation* biozones exhibited the highest mean values, forming a statistically distinct group (*p* < 0.05) from the Savanna and Steppe-Savanna, which had the lowest precipitation levels. This outcome aligns with expected eco-physiographic traits: biozones with denser vegetation or located in regions of greater elevation and slope tend to receive higher rainfall interception and maintain more humid microclimates^65,68^.

In contrast, savanna biozones are mostly located in flatter areas with lower water input, reflecting the typical water limitation of the northeastern Brazilian semiarid region^30^. This variation in precipitation among vegetation types may strongly influence primary productivity, CO_2_ fluxes, and ecological regeneration dynamics, especially in response to disturbances such as fire and deforestation^33^.

Regarding annual mean temperature, significant differences were also observed between biozones. The highest temperatures occurred in the *Steppe-Savanna*, Savanna, and *Contact areas* (ecotones/enclaves), consistent with their location in more exposed areas, characterized by low vegetation cover and high solar radiation incidence. These conditions tend to favor surface heat accumulation and reduced relative humidity, which may enhance carbon emissions through soil respiration and organic matter degradation^15,65^. On the other hand, the *Dense Ombrophilous Forest* and *Open Ombrophilous Forest* biozones exhibited the lowest mean temperatures, statistically differing from the others. This pattern is associated with the shading effect, higher canopy density, and the presence of deeper and moister soils, which function as local microclimatic buffers^62^. The lower mean temperature in these zones suggests a greater capacity for thermal moderation and potentially higher resilience to regional climate change.

These climatic distinctions between biozones help explain part of the variability observed in XCO_2_ anomalies and underscore the importance of considering local climatic contexts when interpreting carbon emission and removal patterns. Moreover, the results provide valuable input for identifying priority areas for mitigation and climate adaptation strategies in the Caatinga biome.

### Correlation patterns between XCO_2_, vegetation, and climate

Partial correlation analyses between XCO_2_, vegetation indices (NDVI, EVI), and climate variables (temperature and precipitation) revealed biome-wide patterns in the Caatinga (Table 3). When statistically controlling for the shared variance among predictors, XCO_2_ exhibited only a weak and non-significant association with temperature and vegetation indices (*p* > 0.05), indicating that short-term phenological or thermal fluctuations do not directly explain atmospheric CO_2_ variability at the biome scale. In contrast, XCO_2_ maintained a significant positive relationship with precipitation (*p* < 0.05), consistent with rainfall-induced shifts in ecosystem carbon exchange via growth stimulation or moisture-driven respiration pulses.

Variance Inflation Factor (VIF) diagnostics revealed extreme multicollinearity between NDVI and EVI (VIF > 36), while climate variables remained within acceptable thresholds. This redundancy among vegetation indices indicates that both metrics capture largely overlapping phenological patterns; however, after accounting for shared variance, NDVI retained the strongest independent relationship with XCO_2_ anomalies, reinforcing its importance as the dominant vegetation-based predictor in the biome.

Overall, the strong dependence of vegetation indices on hydrological seasonality shown by their positive relationship with precipitation and negative association with temperature highlights the overarching role of water availability in regulating ecosystem functioning across the Caatinga. These statistical patterns suggest that the response of atmospheric XCO_2_ to biome processes is subtle and likely modulated by regional atmospheric transport and broader-scale carbon dynamics, rather than being driven solely by local vegetation activity.

**Table 3:** Synthesis of relationships among NDVI, EVI, and environmental variables in the Caatinga.

Study/source	Vegetation types/region	Key relationships with NDVI	Key relationships with EVI	Relevant environmental controls	Notes
This study (2025)	Caatinga (various phytophysiognomies)	Strong positive correlation with EVI (*r* > 0.86). Negative correlation with temperature. Positive correlation with precipitation	Strong positive correlation with NDVI (*r* > 0.86). Negative correlation with temperature. Positive correlation with precipitation	Hydrological seasonality; temperature-driven stress; structural differences among vegetation types	Explains spatial–temporal variability in XCO_2_ anomalies.
Medeiros et al^69^.	Caatinga preserved vs. degraded areas	NDVI strongly driven by precipitation (*r* = 0.72–0.88). Sharp declines during droughts	EVI also tightly linked to rainfall and canopy structure; better captures degradation	Rainfall seasonality; drought intensity; land-use pressure	Shows different sensitivity between preserved and degraded areas.
Zou et al^70^.	Caatinga dry forest sites	NDVI decreases with higher temperatures and VPD; increases with soil moisture and accumulated rainfall	Similar pattern to NDVI but more responsive to canopy density	Soil moisture; air temperature; vapor pressure deficit	Identifies temperature as a limiting factor during dry season.
Barbosa et al^71^.	Shrublands and open Caatinga	NDVI highly seasonal; peaks depend on onset of rains. Weak vegetation activity under prolonged droughts	EVI more stable during early season moisture pulses	Hydrological pulses; early-rainfall events	Highlights differential phenological sensitivity.

## Conclusion

This study revealed that XCO_2_ anomalies in the Caatinga biome were predominantly positive, exhibiting markedly heterogeneous spatial and temporal patterns modulated by climatic, structural, and phenological factors specific to each phytophysiognomy. The combination of spatial autocorrelation analysis, ARIMA time series modeling, and correlations with environmental variables showed that zones such as the *Steppe-Savanna* and *Pioneer Formation* are particularly prone to fluctuations in atmospheric carbon concentrations, with concerning signs of recent increases. The strong dependence of these biozones on hydrological and thermal seasonality, coupled with increasing anthropogenic pressure, suggests that the biome responds acutely to environmental and climatic disturbances, compromising its role as a potential CO_2_ sink.

The presence of emission hotspots and the instability observed in some forest formations point to the growing vulnerability of this ecosystem. In light of intensifying climate change and the historical neglect of Caatinga conservation, the findings presented here reinforce the urgency of targeted actions for monitoring, protection, and ecological restoration of this biome. Protecting the Caatinga is not only essential for preserving its biodiversity and ecosystem services but also for mitigating carbon emissions and contributing strategically to national and global climate goals.

## Data Availability

The datasets generated and/or analysed during the current study are available in the GitHub repository https://github.com/arpanosso/caatinga-xco2-carbon-vulnerability. The XCO_2_ data used in this study were obtained from the Orbiting Carbon Observatory-2 (OCO-2) dataset, available at https://ocov2.jpl.nasa.gov/science/oco-2-data-center/.
